# The Origin Recognition Complex Interacts with a Subset of Metabolic Genes Tightly Linked to Origins of Replication

**DOI:** 10.1371/journal.pgen.1000755

**Published:** 2009-12-04

**Authors:** Erika Shor, Christopher L. Warren, Joshua Tietjen, Zhonggang Hou, Ulrika Müller, Ilaria Alborelli, Florence H. Gohard, Adrian I. Yemm, Lev Borisov, James R. Broach, Michael Weinreich, Conrad A. Nieduszynski, Aseem Z. Ansari, Catherine A. Fox

**Affiliations:** 1 Department of Biomolecular Chemistry, School of Medicine and Public Health, University of Wisconsin, Madison, Wisconsin, United States of America; 2 Department of Biochemistry, College of Agricultural and Life Sciences, University of Wisconsin, Madison, Wisconsin, United States of America; 3 The Genome Center, University of Wisconsin, Madison, Wisconsin, United States of America; 4 Institute of Genetics, Queen's Medical Centre, University of Nottingham, Nottingham, United Kingdom; 5 Department of Mathematics, College of Letters and Science, University of Wisconsin, Madison, Wisconsin, United States of America; 6 Department of Molecular Biology, Princeton University, Princeton, New Jersey, United States of America; 7 Laboratory of Chromosome Replication, Van Andel Research Institute, Grand Rapids, Michigan, United States of America; Stanford University School of Medicine, United States of America

## Abstract

The origin recognition complex (ORC) marks chromosomal sites as replication origins and is essential for replication initiation. In yeast, ORC also binds to DNA elements called silencers, where its primary function is to recruit silent information regulator (SIR) proteins to establish transcriptional silencing. Indeed, silencers function poorly as chromosomal origins. Several genetic, molecular, and biochemical studies of *HMR*-E have led to a model proposing that when ORC becomes limiting in the cell (such as in the *orc2-1* mutant) only sites that bind ORC tightly (such as *HMR*-E) remain fully occupied by ORC, while lower affinity sites, including many origins, lose ORC occupancy. Since *HMR*-E possessed a unique non-replication function, we reasoned that other tight sites might reveal novel functions for ORC on chromosomes. Therefore, we comprehensively determined ORC “affinity” genome-wide by performing an ORC ChIP–on–chip in *ORC2* and *orc2-1* strains. Here we describe a novel group of *orc2-1*–resistant ORC–interacting chromosomal sites (ORF–ORC sites) that did not function as replication origins or silencers. Instead, ORF–ORC sites were comprised of protein-coding regions of highly transcribed metabolic genes. In contrast to the ORC–silencer paradigm, transcriptional activation promoted ORC association with these genes. Remarkably, ORF–ORC genes were enriched in proximity to origins of replication and, in several instances, were transcriptionally regulated by these origins. Taken together, these results suggest a surprising connection among ORC, replication origins, and cellular metabolism.

## Author Summary

Chromosomes must be replicated prior to cell division. The process of duplication of each eukaryotic chromosome starts at discrete sites called origins of replication. An evolutionarily conserved Origin Recognition Complex (ORC) binds origins and helps make them replication-competent. ORC also binds another class of chromosomal sites that primarily function not as origins but as “silencers.” Silencers serve as starting points for the formation of silent chromatin, a special structure that represses local gene transcription in a promoter-independent fashion. One yeast silencer studied in great detail was found to bind ORC *in vitro* and *in vivo* with high affinity (“tightly”). On the other hand, several replication origins were found to bind ORC with lower affinity (“loosely”). We performed a genome-wide comparison of ORC affinity and found a novel class of high-affinity ORC–binding sites. Surprisingly, this class consisted neither of origins nor of silencers but of highly expressed genes involved in various metabolic processes. Transcriptional activation helped target ORC to these sites. These genes were frequently found near origins of replication, and in several instances their transcription was affected by deletion of the nearby origin. These results may shed light on a new molecular mechanism connecting nutrient status and cell division.

## Introduction

In eukaryotes, the process of DNA replication occurs in the context of chromatin and is tightly controlled at multiple levels. Studies of budding yeast *Saccharomyces cerevisiae*, a unicellular eukaryote, have led to crucial insights into the interplay between chromatin structure, gene expression, and DNA replication. In yeast, as in higher eukaryotes, the first step in DNA replication occurs when the Origin Recognition Complex (ORC), an evolutionarily conserved heterohexamer, recognizes and binds sites on the chromosome called origins of replication [1]. During the G1 phase of the cell cycle, ORC recruits additional factors to origins, including the Mcm2-7 replicative helicase, resulting in formation of the pre-replicative complex (pre-RC) [2]. When the cell is ready to start DNA replication, phosphorylation of pre-RC subunits by S phase kinases triggers DNA unwinding at the origins, or origin firing [2]. The decision to enter S phase and initiate DNA replication is regulated by cell size and nutritional status via molecular mechanisms that are still imperfectly understood [3].

Budding yeast replication origins are predominantly located in intergenic spaces, presumably to separate the processes of replication initiation and transcription. The few exceptions to this rule are origins that are either located within meiosis-specific genes, whose transcription is repressed in mitotically growing cells [4], or origins that are inactive during normal growth when the gene is expressed (e.g. *ARS604*; [5],[6]). The idea that transcription interferes with replication initiation is also supported by the observation that origins located downstream of protein-coding genes are more sensitive to mutations in pre-RC components and that high levels of transcription across an origin impair its function [7],[8]. Each known yeast origin is given an ARS (autonomous replication sequence) name followed by a number that usually reflects its chromosomal position. Unlike origins in higher eukaryotes and fission yeast, *S. cerevisiae* origins contain an ORC-binding motif with a discernible ARS consensus sequence (ACS) that is necessary but not sufficient for ORC binding [9],[10].

Several studies aiming to comprehensively identify yeast origins have employed microarray-based methods to find sites of pre-RC binding or replication bubble formation throughout the genome [11]–[15]. A large number of studies has also examined origins directly either on the chromosome (by two-dimensional gel electrophoresis) or in plasmid-based assays. These studies have demonstrated that different origins are programmed to fire at different times during S phase and with varying efficiency (proportion of cell cycles in which the origin fires; [16],[17]). Early origin firing time often correlates with higher origin efficiency, while late firing origins are usually less efficient. Some very late and inefficient origins may never fire on the chromosome, but when analyzed on plasmids in isolation of other origins, they are able to fire and promote plasmid replication [5],[6],[18]. The wealth of information gathered from both individual and genome-wide origin studies has been systematically summarized in the DNA Replication Origin Database, OriDB (www.oridb.org; [19]). Here, sites for which origin activity has been demonstrated either on the chromosome or on a plasmid have been annotated as “confirmed” ARSs. Sites identified in two or more microarray-based studies but without direct confirmation of origin activity were classified as “likely” ARSs, while sites identified in only one microarray study were named “dubious” ARSs.

OriDB lists over 700 ORC sites, compared to 300–400 actively firing origins, suggesting that many ORC sites either function extremely inefficiently as replication origins or have other functions. Indeed, one additional role for ORC sites is well established: they can function as silencers, or sites where formation of silent chromatin is initiated [20]. Budding yeast has silent chromatin at two types of loci: silent mating type loci (*HMR* and *HML*) on chromosome III and telomeres [21],[22]. The *HMR*-E silencer is both necessary and sufficient to establish silent chromatin at *HMR* [20]. *HMR*-E contains an ORC binding site but in contrast to replication origins, ORC binding there does not lead to efficient origin firing [23],[24], instead helping recruit silent information regulator (SIR) proteins, resulting in spreading of silent chromatin across *HMR* [25]. Like heterochromatin in higher eukaryotes, silent chromatin is characterized by highly compacted, hypoacetylated nucleosomes and by suppression of both transcription and replication initiation [26].

Studies of conditional alleles of the essential genes encoding pre-RC components have provided many important insights into replication initiation. One ORC allele frequently used in these studies is *orc2-1*. The major molecular defect in *orc2-1* cells is reduction of Orc2p levels and stability of ORC as a whole, even at the permissive temperature [27],[28]. Interestingly, origin firing at *HMR*-E is enhanced in the *orc2-1* mutant relative to the wild type strain [24]. This behavior may be unique to *HMR*-E, as firing from several other replication origins decreases in the *orc2-1* mutant [29]. *HMR*-E also exhibits unique behavior *in vitro*, where it binds purified ORC with very high affinity [23]. To explain the unusual behavior of *HMR*-E both *in vivo* and *in vitro*, it has been proposed that the *orc2-1* mutant reduces the levels of functional ORC such that only those sites that bind ORC tightly, e.g. *HMR*-E, remain fully occupied by ORC [23]. On the other hand, lower affinity sites, such as those at several non-silencer origins, are not fully occupied by ORC in the *orc2-1* strain and therefore exhibit reduced origin firing. Because firing from nearby origins is decreased, *HMR*-E is not as frequently replicated by a passing replication fork and gets a chance to fire, thus explaining increased firing from *HMR*-E in the *orc2-1* mutant. Thus, *orc2-1* resistance or sensitivity can serve as an indicator of high or low affinity for ORC, respectively. Since there is an example of an *orc2-1*-resistant ORC-binding site, *HMR*-E, whose primary role is distinct from origin firing, we decided to use the *orc2-1* mutation as a tool to comprehensively search for *orc2-1*-resistant ORC sites across *S. cerevisiae* genome. To this end, we performed chromatin immunoprecipitation with ORC antibodies followed by microarray analysis (ChIP-on-chip) in the *ORC2* and *orc2-1* strains. Remarkably, we identified an *orc2-1*-resistant class of ORC-interacting sites distinct from both origins and silencers. Instead, this class of sites mainly consisted of protein-coding genes that were highly expressed, functioned in various metabolic pathways, and were frequently located downstream of replication origins.

## Results

### ORC binding to *HMR*-E *in vivo* was *orc2-1*-resistant

**Figure 1:**
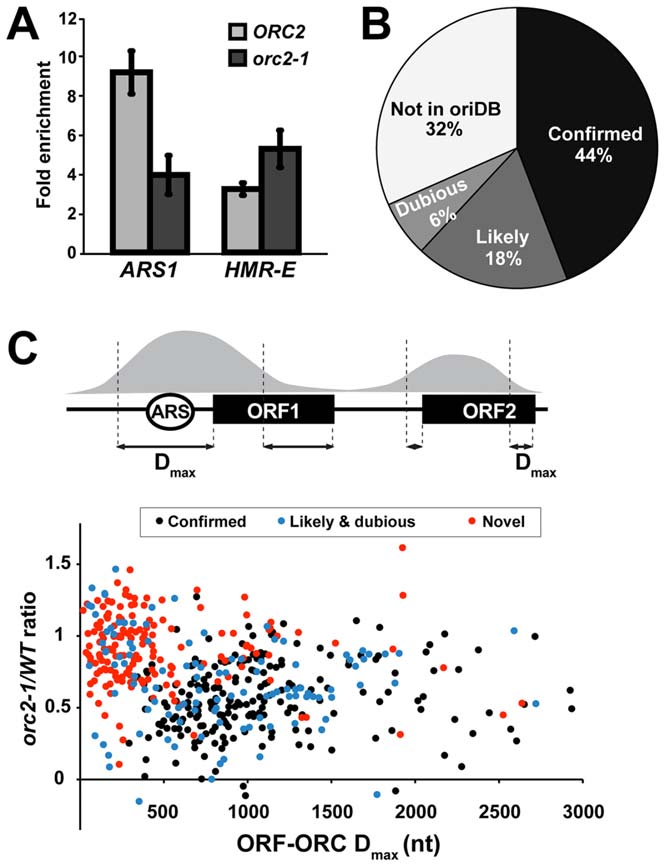
Resistance to *orc2-1* defined a novel class of ORC binding sites. (A) ORC binding to *HMR*-E *in vivo* is *orc2-1*-resistant. ORC ChIPs were performed in the *ORC2* and *orc2-1* strains at the permissive temperature of 23°C, and relative enrichment of *HMR*-E- or *ARS1*-containing DNA fragments was measured by PCR. *ADH4* signal was used as a measure of background ORC binding. (B) The majority of sites identified in our ORC ChIP–on–chip have already been annotated by the Origin Database, validating our identification of *bona fide* ORC sites. (C) Novel ORC sites form an *orc2-1*-resistant, ORF–overlapping cluster. Each ORC peak that overlapped at least one ORF was included in the calculation outlined the diagram. D_max_ was calculated as described in the text and plotted against *orc2-1/WT* ratio. ORC peaks in the graph are classified according to their OriDB annotations. “Novel” sites are those identified for the first time in this study as ORC–binding sites.

To assess the efficiency of ORC binding to *HMR*-E in the *orc2-1* mutant, we immunoprecipitated formaldehyde-crosslinked chromatin fragments from a wild type and an *orc2-1* strain with a cocktail of four monoclonal antibodies against Orc1p, -2p, -3p, and -4p. Relative enrichment of *HMR*-E (containing a high affinity ORC site) and a control origin, *ARS1* (containing a low affinity ORC site), in the precipitated DNA was measured by PCR. We found that binding of ORC to *HMR*-E was resistant to the *orc2-1* mutation, while ORC binding to *ARS1* was reduced by about two-fold (Figure 1A). Thus high affinity binding of ORC to a genomic site helps maintain ORC at that site in a strain where ORC levels are compromised. Therefore, we performed ChIP-on-chip to compare ORC binding in *orc2-1* and wild type strains: sites remaining fully occupied by ORC in the *orc2-1* mutant would be considered high-affinity sites *in vivo*.

### The ORC ChIP–on–chip: an overview

ORC ChIP was performed in a wild type and an *orc2-1* strain as outlined above, and the immunoprecipitated DNA was labeled and hybridized to high density tiled microarrays representing the entire yeast genome. Genome-wide ORC binding in the wild type and mutant strains is shown in Figure S1. To focus on the most robust ORC-binding sites, we chose to study peaks that had been assigned a p-value of 10^−20^ or better by the peak finding software ChiPOTle (Figure S2; Materials and Methods). At this p-value threshold we identified 541 ORC peaks, the majority of which (370, or 68%) had already been identified as ORC sites and annotated in OriDB (Figure 1B; Table S1), validating our ability to detect ORC binding sites with our antibodies. The area of an individual peak was deemed to reflect efficiency of immunoprecipitation of corresponding DNA fragments by the ORC antibodies. Consequently, for every peak, we determined the ratio of its area in the *orc2-1* strain to that in the *ORC2* strain. This *orc2-1/WT* ratio served as the indicator of a site's *orc2-1* sensitivity. Importantly, *orc2-1*/*WT* ratios of *ARS1* (*ARS416*) and *HMR-E* (*ARS317*) were 0.55 and 1.0, respectively, in good agreement with our directed ChIP results.

### New ORC binding sites showed close overlap with open reading frames (ORFs)

We identified 171 peaks not previously observed as sites of ORC binding (Figure 1B). Interestingly, many of these new ORC peaks possessed the following properties: they were resistant to the *orc2-1* mutation and overlapped closely with open reading frames (ORFs) of protein-coding genes. To measure the overlap of ORC peaks with neighboring ORFs, we performed the following analysis (Figure 1C). 91% of ORC peaks in our dataset overlapped with at least one ORF. For every such peak, we picked one ORF with which it had the greatest degree of overlap and measured the two distances between their ends (Figure 1C). When assigning a single ORF to a peak, verified and uncharacterized ORFs, as annotated by the *Saccharomyces* Genome Database (SGD, www.yeastgenome.org), were always chosen over dubious ones. Close overlap between ORC peak and ORF resulted in the end-to-end distances being “small”, while poor overlap resulted in at least one of the two distances being “large” (Figure 1C). Plotting the peaks' *orc2-1*/*WT* ratios versus the larger of the two distances (D_max_) revealed a cluster of ORC sites characterized by *orc2-1* resistance (*orc2-1/WT* ratios near one) and close overlap with ORFs (D_max_<500 nt). In contrast, “confirmed” ARSs (black dots) had large D_max_ values, reflecting poor overlap with ORFs and lower *orc2-1/WT* ratios (Figure 1C). For the rest of the manuscript, we will refer to verified and uncharacterized ORFs (n = 163) that overlapped an ORC peak with a D_max_ of less than 500 nt as “ORF-ORC” (Table S2). Significantly, approximately one-third of the ORF-ORC set was comprised of sites already annotated in OriDB, predominantly as “likely” and “dubious” ARSs (Figure 1C, blue dots; Table S2). Moreover, about thirty of these OriDB ORF-ORC sites were shown to interact with Mcm2 as well as ORC, suggesting that other components of the pre-RC may associate with these sites *in vivo* (Table 1; Table S2) [13]. Interestingly, most ORC peaks corresponding to these ORF-ORC OriDB sites were *orc2-1*-resistant, similarly to the majority of new intra-ORF ORC-binding sites identified by our ChIP-on-chip. Thus, ORC association with protein-coding regions has been observed previously, but here we report the first identification of such non-canonical ORC-interacting sites *en masse* and their classification by *orc2-1* resistance.

**Table 1:** ORF–ORC sites did not function as replication origins on plasmids.

Clone^1^	Peak type	ARS
*ARS731.5*	ORC-MCM^3^	**+**
*TEF2* 2	ORC-MCM^3^	**−**
*CDC48* 2	ORC^4^	**−**
*YRA1*	ORC^4^	**−**
*ACT1*	ORC-MCM^3^	**−**
*TDH3*	ORC-MCM^3^	**−**
*ENO1* 2	ORC-MCM^3^	**−**
*ENO2*	ORC-MCM^3^	**−**
*TDH1* 2	ORC-MCM^3^	**−**
*GPM1*	ORC-MCM^3^	**−**
*FBA1* 2	ORC-MCM^3^	**−**
*UTH1*	ORC-MCM^3^	**−**
*RPL10*	ORC^4^	**−**
*ILV5*	ORC-MCM^3^	**−**
*RPL3*	ORC-MCM^3^	**−**
*TEF1* 2	ORC-MCM	**−**
*ASN1* 2	ORC-MCM^3^	**−**

1 Chromosomal coordinates of all clones can be found in Table S3.

2 ORF-ORC genes for which promoter-less constructs were also tested.

3 Genes where both ORC and MCM bind within the ORF [14].

4 This study.

**Figure 2:**
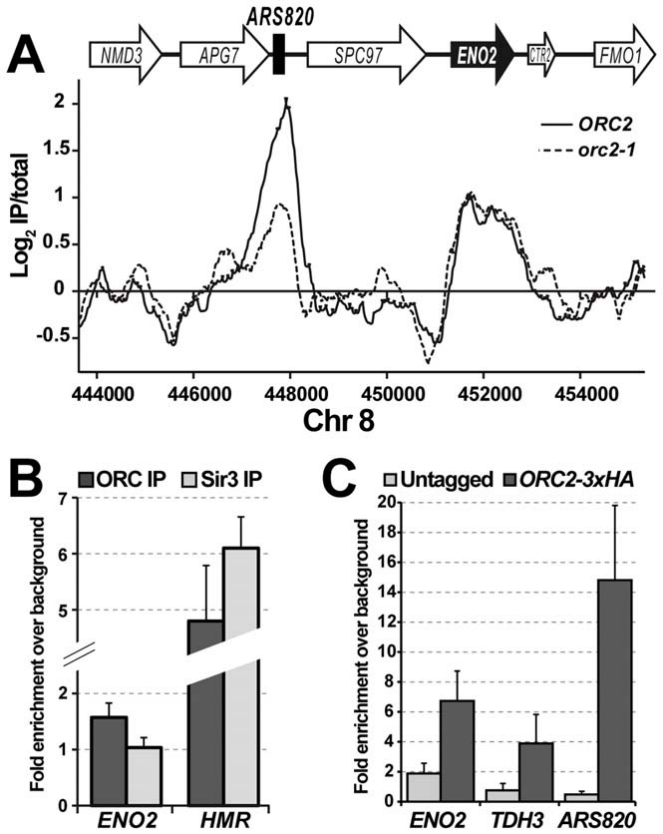
Detection of ORF–ORC sites in directed ChIP experiments. (A) Examples, from the ChIP–on–chip data, of an *orc2-1*-sensitive ORC peak at a replication origin, *ARS820*, and of a nearby *orc2-1*-resistant ORF–ORC peak at the *ENO2* gene. (B) Immunoprecipitation with a cocktail of monoclonal antibodies against Orc1p, Orc2p, and Orc3p resulted in a slight enrichment of the *ENO2* ORF relative to a background locus (*FKH1*), while immunoprecipitation with a cocktail of two monoclonal Sir3 antibodies did not result in any enrichment of *ENO2*. On the other hand, the Sir3 antibodies, similarly to the ORC antibodies, were able to efficiently immunoprecipitate *HMR*. (C) ChIPs with an anti–HA antibody were performed in Orc2-3xHA and untagged strains. Association of the antibody with two different ORF-ORC loci, *ENO2* and *TDH3*, as well as an origin of replication, *ARS820*, was dependent on the presence of HA–tagged Orc2. All data were normalized to immunoprecipitated *FKH1* from the same sample. Averages of two biological replicates of each strain are plotted, with error bars representing one standard deviation.

The ORF-ORC peaks were generally more shallow and uniform in shape than replication origin ORC peaks. Figure 2A shows an example of an *orc2-1*-sensitive origin (*ARS820*) next to a “likely” ARS with a typical ORF-ORC peak – *orc2-1*-resistant, shallow and uniform in shape, and overlapping closely with the ORF of *ENO2*. Figure S3 shows ORC and Mcm2 binding across the same region from the ChIP-on-chip study done by Xu et al [13], demonstrating that an independently derived set of ORC antibodies, an unrelated Mcm2 antibody, and a different set of genomic arrays have detected ORC and MCM binding throughout *ENO2* ORF. Since tiled arrays are likely to be more sensitive detectors of binding than directed ChIPs [30], we suspected that the ORF-ORC peaks would be relatively difficult to detect in directed ChIPs. Indeed, for the several ORF-ORC sites tested in directed ChIPs, we observed approximately 1.5 to two-fold enrichment over background in contrast to five-fold and greater enrichment for ARS sites (Figure 2B, see more examples below). This small enhancement is likely significant, however, because a cocktail of two monoclonal Sir3 antibodies failed to give any enrichment over background at *ENO2* but could efficiently immunoprecipitate *HMR* (Figure 2B).

To ascertain further that the ORF-ORC sites were enriched in the ORC IPs due to their association with ORC and not to a non-specific antibody association or another ChIP artifact, we tagged Orc2 on the C-terminus with a triple HA epitope tag at its endogenous chromosomal locus. The Orc2-3xHA fusion protein was functional as judged by the strain's viability and normal growth rate (data not shown). We then performed ChIPs with an HA antibody in the tagged and untagged strains. Indeed, we found that two different ORF-ORC sites were efficiently immunoprecipitated in the Orc2-3xHA strain but not in the untagged strain (Figure 2C), indicating that ORC was associating with these chromosomal sites *in vivo*.

### ORF–ORC sites did not function as origins of replication

**Figure 3:**
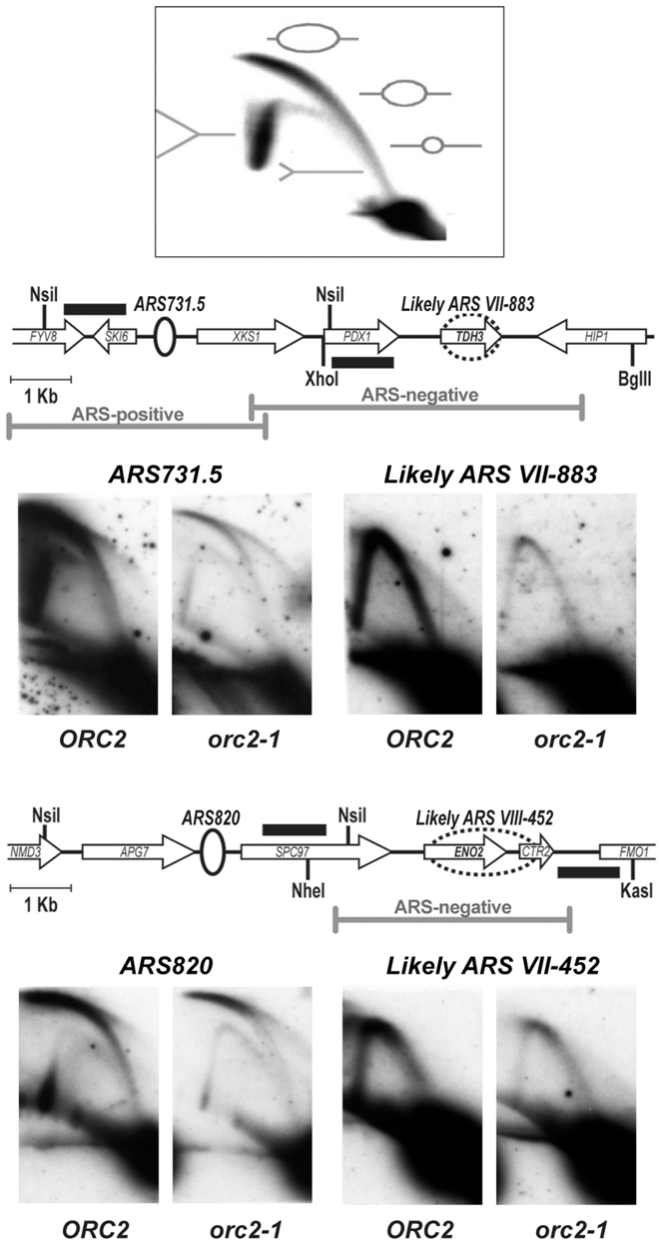
ORF–ORC sites did not function as origins of replication on the chromosome. The diagram shows expected migration patterns of different replication intermediates (replication bubbles and forks) on 2D gels. The ratio of bubbles to small forks corresponds to origin efficiency. Restriction enzyme and probe positions (black rectangles) are shown. The two “confirmed” origins, *ARS731.5* and *ARS820*, are *orc2-1*-sensitive (with *orc2-1/WT* ratios of 0.36 and 0.35, respectively), corresponding to a decrease in firing at these sites in the *orc2-1* mutant. Both ORF–ORC sites, *TDH3* and *ENO2*, have been detected as sites of ORC and MCM binding in another ChIP–based study [13] and are annotated as “likely” ARSs by OriDB. In the figure, dashed ovals indicate “likely” ARS boundaries. Both “likely” ARSs at *TDH3* and *ENO2* are *orc2-1*-resistant (*orc2-1/WT* ratios of 1.1 for each), but neither one produced replication bubbles either in the wild-type or in the *orc2-1* strain. Gray bars indicate chromosomal clones tested in plasmid origin assays (Table 1, Table S3).

As mentioned above, the ORF-associated ORC peaks were generally *orc2-1*-resistant (Figure 1C, Figure 2A). This property made us wonder whether, similar to *HMR*-E, ORF-ORC sites could serve as “back-up” origins under “low ORC” conditions that strongly affect other origins (e.g. in the *orc2-1* mutant). To test this hypothesis, we performed 2D gel assays in the *ORC2* and *orc2-1* strains on two pairs of neighboring ORC sites: “confirmed” *ARS820* – “likely” *ARSVIII-452*/*ENO2* and “confirmed” *ARS731.5* - “likely” *ARSVII-883*/*TDH3* (Figure 3). Both “confirmed” ARSs were *orc2-1*-sensitive for ORC binding and for firing: intensity of the bubble arc relative to that of the short fork arc decreased at these origins in the *orc2-1* mutant. At the *orc2-1*-resistant “likely” ARSs (the ORF-ORC sites) we were unable to detect bubble arcs either in the wild type or in the *orc2-1* strains, suggesting that these sites did not fire under normal laboratory growth conditions, nor did they become more active in the *orc2-1* mutant, in contrast to *HMR*-E [24].

Some origins exist that do not fire on the chromosome but can fire on a plasmid, helping maintain the plasmid in the cell [5],[6],[18]. To ask whether ORF-associated ORC sites could in principle function as origins of replication, we cloned chromosomal fragments containing 16 ORF-ORC sites onto plasmids and asked whether these clones could transform yeast cells. We focused primarily on ORF-ORC sites that had been annotated in OriDB and where the ORF had been shown to associate with Mcm2 protein, reasoning that these had the highest likelihood of possessing origin activity (Table 1; [13]). We also tested a number of intergenic ORC peaks in the same ARS assay, with “confirmed” *ARS731.5* serving as our positive control. We found that while the majority of intergenic ORC peaks did possess ARS activity, none of the ORF-ORC peaks did (Table 1; for full ARS assay results see Table S3).

There are a few documented examples of intra-gene origin firing in budding yeast [4]–[6]. In one of these cases, the gene is not transcribed during mitotic growth and is induced only during meiosis, so origin firing and transcription are temporally separated [4]. Since none of the ORF-ORC genes were meiotic (Table S2), it seemed unlikely that a similar mechanism was operating at these loci. However, since the ChIP signal was over the ORF at these sites, we tested whether abolishing transcription would promote origin firing from within the ORF. Thus, we tested several ORF-ORC clones lacking the gene's promoter in the ARS assay. We saw that even promoter-less ORC-binding ORFs were unable to promote plasmid maintenance (Table 1; Table S3). We concluded that ORC-ORF sites could not function as replication origins, raising the possibility that ORC association with them might reflect a novel function for ORC.

A shared feature of all yeast origins of replication is the ARS Consensus Sequence (ACS)—an AT-rich sequence necessary but not sufficient for ORC binding [9],[31]. We employed MEME motif-finding software (http://meme.sdsc.edu) to search for the ORC binding motif within the ORF-ORC set and did not identify one. Nor did we identify other consensus motifs with any degree of significance. We concluded that ORC binding to these sites was probably mediated by a mechanism distinct from that operating at canonical replication origins. The more shallow and uniform shape of the ORF-ORC peaks also supported the idea that ORC interacted not with a single sequence within the ORF, but all along the ORF (e.g. with chromatin components or RNA). Thus, ORF-ORC sites were likely distinct from replication origins in terms of both structure and function.

### “Confirmed” origins could be both *orc2-1*-resistant and efficient

**Figure 4:**
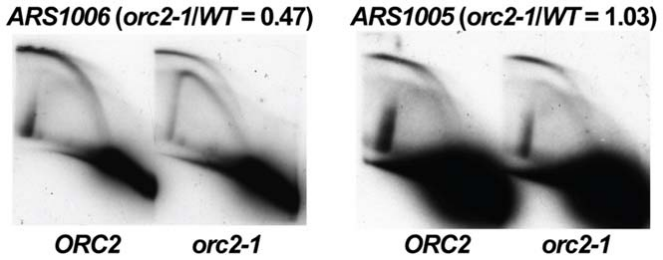
Origins of replication could combine *orc2-1*-resistance with high firing efficiency. Unlike *HMR*-E, *orc2-1*-resistant replication origin *ARS1005* could fire with high efficiency at its endogenous chromosomal location. Like *HMR*-E, *ARS1005* was resistant to *orc2-1* for firing efficiency, while neighboring origin *ARS1006* that was *orc2-1*-sensitive for ORC binding was also *orc2-1*–sensitive for firing.

As described above, we identified a large number of *orc2-1*-resistant sites that did not function as origins of replication. Similarly, *HMR*-E is *orc2-1*-resistant and a very inefficient origin [23],[24]. Consistent with these observations, the majority of “confirmed” origins were more *orc2-1*-sensitive than *HMR*-E or ORF-ORC sites (Figure 1C). However, we did identify a number of *orc2-1*-resistant origins (Figure 1C, black dots with *orc2-1/WT* ratios near one). We analyzed origin firing by 2D gel electrophoresis at several *orc2-1*-sensitive and -resistant “confirmed” origins in the *ORC2* and *orc2-1* strains, with a representative pair shown in Figure 4. One observation readily made from these replication assays was that origins of replication could combine *orc2-1* resistance with high firing efficiency (Figure 4, *ARS1005*). We also observed a correlation between how efficiently ORC bound to an origin in the *orc2-1* mutant and how well the origin fired in that mutant. In other words, origins with lower *orc2-1*/*WT* ratios (below 0.5) fired less efficiently in the *orc2-1* mutant than in the wild type strain (Figure 4, *ARS1006*; see also *ARS731.5* and *ARS820* in Figure 3). From this line of investigation we concluded that, in contrast to *HMR*-E and the ORF-ORC sites, non-silencer origins could combine high affinity for ORC with efficient origin firing.

### Transcription promoted ORC association with protein-coding regions

**Figure 5:**
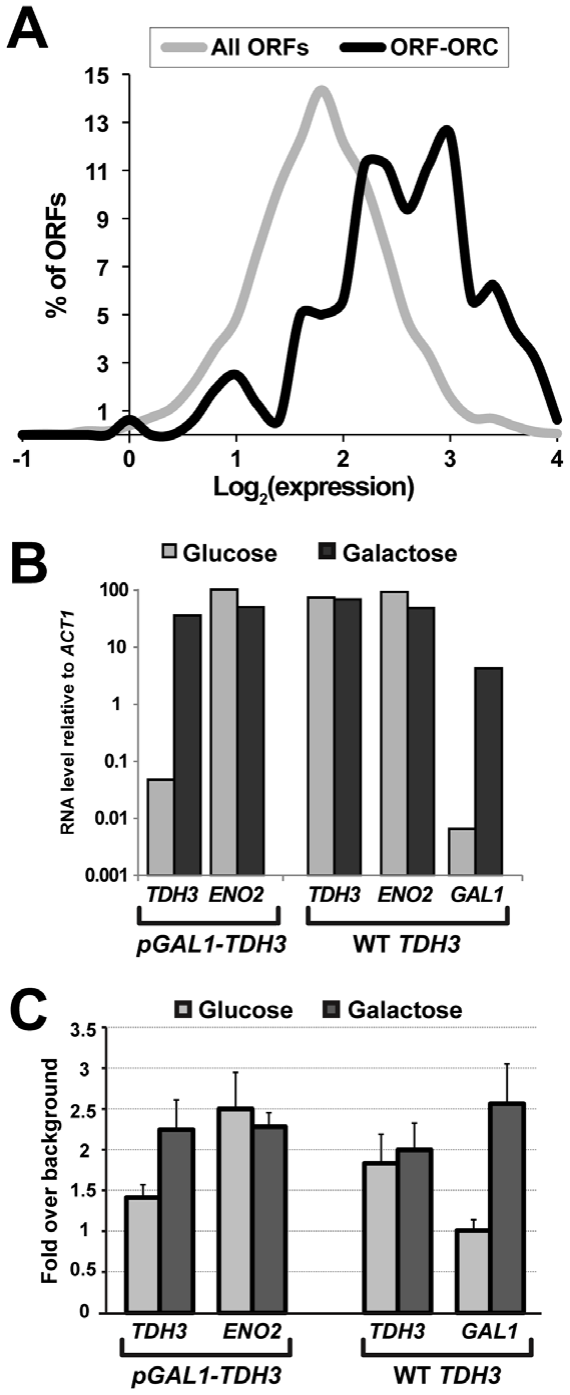
Transcriptional activity promoted ORC binding to an ORF. (A) Histograms of expression levels of the ORF–ORC set and of all genomic ORFs are shown. Dubious ORFs were not included in this analysis. (B) *TDH3* promoter was replaced by the *GAL1* promoter (p*GAL1*). p*GAL1-TDH3* and wild-type strains were grown in glucose or galactose, RNA was isolated, and gene expression measured by reverse transcriptase PCR. Growth in glucose repressed *TDH3* transcription in the p*GAL1-TDH3* strain but not in an isogenic wild-type strain, whereas wild-type *TDH3* was virtually unaffected by change of carbon source. As expected, expression of the *GAL1* gene was repressed in glucose and induced in galactose. *ENO2* expression was monitored as a control. (C) Results of directed ORC ChIPs on the wild-type and p*GAL1-TDH3* strains grown in glucose and galactose are shown. Averages of two to four independent biological replicates are plotted for each condition, with error bars representing one standard deviation. Repression of p*GAL1-TDH3* transcription by glucose reduced ORC binding to *TDH3* ORF, while induction of *GAL1* in galactose increased ORC binding there by 2.5-fold.

Since we identified a large number of ORC-interacting sites that did not function as origins of replication or possess the canonical ORC binding motif, we looked for common properties of ORC-binding ORFs to begin to address their functional significance. For these bioinformatical analyses, the entire ORF-ORC set was used, including sites previously listed in OriDB and new sites identified by our ChIP-on-chip. Using the SGD Gene Ontology Slim Mapper (http://www.yeastgenome.org/cgi-bin/GO/goTermFinder.pl) we found that genes involved in various metabolic processes were highly over-represented among genes in the ORF-ORC set (Table 2). Since these genes are involved in metabolizing different kinds of nutrients, such as sugars and amino acids, we wondered if they might be highly expressed under conditions when cells are harvested for ChIP (exponential growth in rich glucose medium). For a global look at gene expression, we analyzed microarray data where RNA had been isolated from wild type cells grown in rich glucose medium and hybridized to the same high density tiled arrays as used for the ORC ChIP-on-chip (Tietjen et al, unpublished). Indeed, comparing expression profiles of all yeast protein-coding genes to the ORF-ORC genes revealed that the ORF-ORC set was largely comprised of highly expressed genes (Figure 5A). However, high transcription was not sufficient to cause ORC binding: although many highly expressed genes were bound by ORC, an even larger number expressed to similar levels were not (Figure S4).

**Table 2:** Metabolic process genes were highly over-represented in the ORF–ORC set.

Gene Ontology Term	P-value^1^
carboxylic acid metabolic process	1.59E-16
organic acid metabolic process	1.59E-16
amino acid biosynthetic process	1.47E-11
amine biosynthetic process	7.77E-11
nitrogen compound biosynthetic process	9.46E-11
cellular amine metabolic process	8.99E-10
cellular nitrogen compound metabolic process	1.26E-09
amino acid metabolic process	1.83E-09
nitrogen compound metabolic process	1.93E-09
cellular amino acid and derivative metabolic process	2.47E-09
generation of precursor metabolites and energy	6.92E-07
monocarboxylic acid metabolic process	1.29E-06
glycolysis	1.48E-06
glucose catabolic process	4.10E-06
hexose catabolic process	1.96E-05
pyruvate metabolic process	3.41E-05
monosaccharide catabolic process	7.34E-05

1 Generated by SGD GO term finder (http://www.yeastgenome.org/cgi-bin/GO/goTermFinder.pl).

To test directly whether high transcriptional state helped promote ORC association with an ORF *in vivo*, we replaced the promoter of one ORF-ORC gene, *TDH3*, by the *GAL1* promoter, inducible by galactose and repressible by glucose. *TDH3*, which encodes a glycolysis and gluconeogenesis protein glyceraldehyde-3-phosphate dehydrogenase, is a highly expressed gene under standard laboratory growth conditions. As expected, growing the *pGAL1-TDH3* strain in glucose repressed *TDH3* expression over 100-fold, while growing it in galactose induced it to levels slightly lower than, but comparable to *TDH3* driven by its native promoter (Figure 5B). As controls, we also measured expression levels of another ORF-ORC gene, *ENO2*, and of the native *GAL1* gene. *ENO2* expression decreased very slightly in galactose compared to glucose, while *GAL1* expression, as predicted, was highly induced by galactose and repressed by glucose (Figure 5B). Next, we performed ORC ChIPs on both *pGAL1-TDH3* and wild type strains grown in glucose or galactose, with the results summarized in Figure 5C. We observed that repression of *GAL1*-driven *TDH3* transcription in glucose reduced ORC association with *TDH3* ORF (1.47-fold enrichment over background in glucose versus 2.24-fold enrichment in galactose, p-value = 0.007). Growth in galactose did not affect ORC association with wild type *TDH3* or to *ENO2*. Interestingly, growth in galactose caused a striking increase in ORC ChIP signal at *GAL1* ORF: from background levels in glucose to a 2.5-fold increase over background in galactose. These results strongly suggested that some aspect of high transcriptional state facilitated ORC association with the ORF-ORC sites. It is notable, however, that even when *TDH3* was transcriptionally repressed, ORC association with its ORF was still approximately 50% above background (Figure 5C). Taken together with the observation that many highly transcribed genes did not associate with ORC (Figure S4), this result indicated that gene properties in addition to transcription promoted their interaction with ORC.

Azvolinsky and colleagues have recently reported that both the leading strand polymerase and another component of the replisome, the Rrm3 DNA helicase, associated with highly transcribed ORFs in *S. cerevisiae*, and that this association was transcription-dependent [32]. We compared our data to those of Azvolinsky et al and found that 38 ORFs that associated with ORC in our study also interacted with DNA polymerase and/or Rrm3 (Table S2). Thus, multiple components of the replication apparatus, including pre-RC factors and replication fork-associated proteins, interact with highly transcribed genes.

### ORF–ORC sites are enriched downstream of “confirmed” ARSs

**Figure 6:**
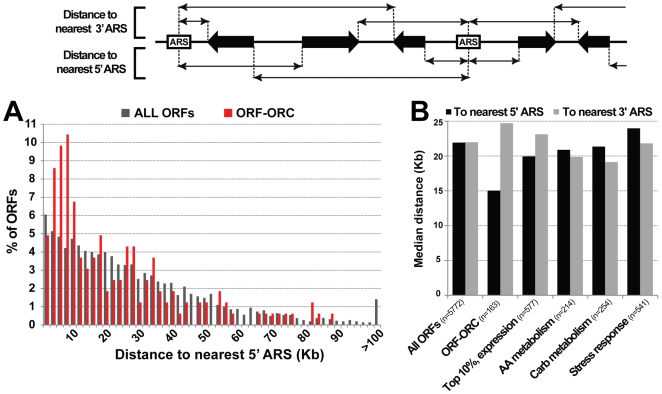
ORF–ORC sites are enriched downstream of replication origins. The schematic at the top of the figure shows how distances to the nearest 5′ and 3′ ARSs were calculated for every ORF in the genome (dubious ORFs were omitted from this analysis). (A) The ORF–ORC set was enriched for genes located within 10 Kb of the nearest upstream origin compared to all ORFs. (B) Median distances to nearest 5′ and 3′ origins are plotted for several classes of ORFs, categorized by expression level or functional process. The ORF–ORC set was the only gene category closely associated with upstream origins.

We observed that many ORF-ORC genes were lying downstream of confirmed origins of replication (e.g. *ENO2* downstream of *ARS820* and *TDH3* downstream of *ARS731.5*, Figure 3). To quantify this observation, for every verified and uncharacterized ORF in the genome we calculated the distance from its 5′ end (the start codon) to the center of the nearest upstream “confirmed” ARS, as well as the distance from its 3′ end (the stop codon) to the center of the nearest downstream “confirmed” ARS (Figure 6). ARS centers were defined as the midpoints of OriDB-annotated ARS regions. We found that genes within the ORF-ORC set were almost twice as likely to reside within 10 Kb of the nearest upstream ARS as a non-ORC binding gene (Figure 6A). The median distance to the nearest upstream ARS was 15.0 Kb for the ORF-ORC set and 21.9 Kb for all genes (p-value = 0.0007). In contrast, distances to the nearest downstream ARS were slightly greater for the ORF-ORC set than for all genes (medians of 24.7 Kb versus 22.0 Kb, respectively).

Since many of the genes within the ORF-ORC set were highly expressed and/or functioned in various metabolic processes, we checked whether either of these properties alone could account for their being positioned downstream of origins of replication. Thus, we calculated ORF-ARS distances for the following groups of genes: top 10% most highly expressed genes, amino acid metabolism genes, and carbohydrate metabolism genes. We also performed this analysis for genes involved in stress response because certain stress response genes that are induced by the *orc2-1* mutation are enriched near origins of replication [33]. We found that none of these categories was enriched for genes located in the vicinity of origins (Figure 6B). We concluded that being positioned downstream of and in close proximity to an origin of replication was a unique property of genes that associated with ORC.

### ORF–ORC transcription was modulated by the nearby replication origin

**Figure 7:**
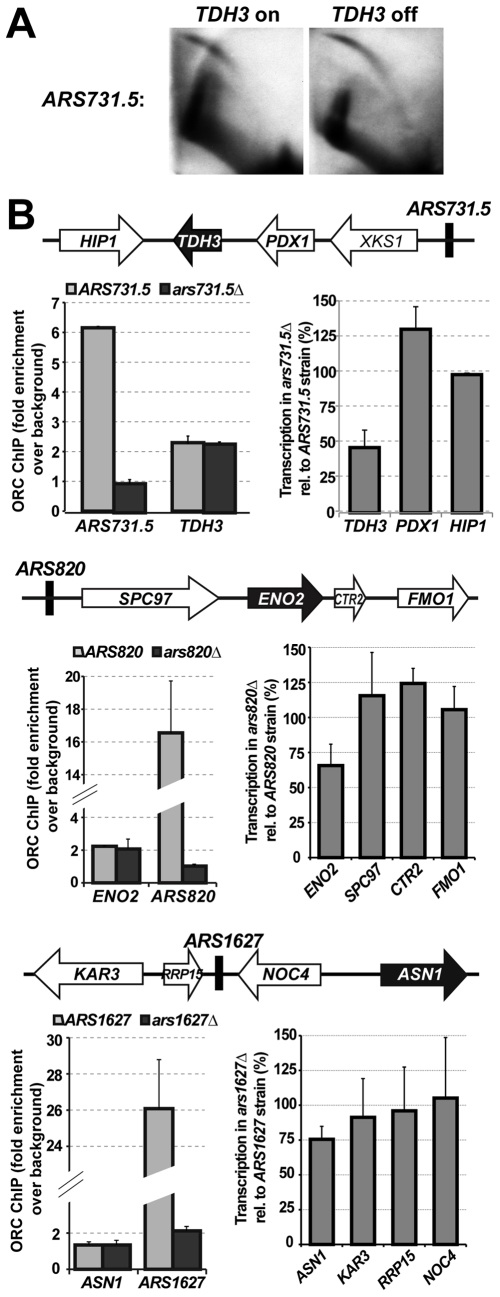
Deletion of a replication origin reduced expression of a downstream ORF–ORC. (A) Repressing *TDH3* transcription by growing the p*GAL1-TDH3* strain in glucose did not grossly alter firing efficiency of the nearby replication origin *ARS731.5* compared to a wild-type strain with a highly transcribed *TDH3* gene. (B) ORC–binding sites within three different replication origins were deleted separately, and both ORC association and gene transcription within the three regions were analyzed. The diagrams show relative positions of genes around the origins. The ORF–ORC genes are shown as black arrows with white font. In each case, deleting the origin's ORC binding site abolished ORC association with the origin but not ORC association with the nearby ORF–ORC. Also in each case, deletion of a replication origin reduced transcription of the nearby ORF–ORC gene while leaving other surrounding genes relatively unaffected. The averages of at least two biological replicates are plotted on the graph, with error bars representing one standard deviation. For the experiment in first panel (*ARS731.5-TDH3* region), quantification of expression of genes near *ARS731.5* was done using band densitometry. For panels 2 and 3, quantification of expression of genes near *ARS820* and *ARS1627* was done using real-time PCR (Materials and Methods).

Proximity of ORF-ORC genes to origins of replication raised the possibility that the origins were regulating these genes and/or vice versa. To test whether expression of an ORF-ORC gene influenced firing efficiency of a nearby origin, we compared *ARS731.5* firing in the wild-type strain to that in the p*GAL1-TDH3* strain. Both strains were grown in glucose, so wild type *TDH3* was highly expressed while p*GAL1-TDH3* transcription was repressed (Figure 5B). The 2D gel origin assay did not reveal any strong effects of *TDH3* transcription on *ARS731.5* efficiency; however, we cannot rule out more subtle differences in origin firing or general replication dynamics within the region (Figure 7A).

To ask the converse question – whether origins could regulate the ORF-ORC genes – we deleted ORC binding sites within three origins of replication, *ARS731.5*, *ARS820*, and *ARS1627*, and measured both ORC association with the nearby ORF-ORC genes (*TDH3*, *ENO2*, and *ASN1*, respectively) and their expression in the deletion mutants (Figure 7B). We found that in all three cases, deletion of the ORC binding site within a replication origin completely abolished ORC binding to the origin, as expected, but did not affect ORC association with the nearby ORF-ORC gene (Figure 7B). We also harvested RNA from wild type and *arsΔ* cells and performed reverse transcriptase PCR to analyze expression levels of the ORF-ORC genes, as well as several other genes in the vicinity of the deleted origin. Surprisingly, we found that transcription of the ORF-ORC genes was reduced by origin deletions while other genes in the region were relatively unaffected (Figure 7B).

## Discussion

### Replication initiation proteins interact with protein-coding regions *in vivo*

In this study, we performed a ChIP-on-chip in the wild type and *orc2-1* mutant strains in order to identify high and low affinity ORC binding sites throughout the yeast genome. We discovered a novel class of *orc2-1*-resistant ORC-associated sites that was distinct from replication origins in several respects: these sites could not initiate replication on the chromosome or a plasmid, did not contain the canonical ORC binding motif, and were localized within protein coding regions of RNA Polymerase II (RNAPII)-transcribed genes. ORC-interacting genes (the ORF-ORC set) had several notable characteristics. They were highly expressed during growth in rich glucose medium, and transcriptional induction promoted, while down-regulation of transcription reduced, ORC interaction with these genes. We also found that these genes were frequently located downstream of replication origins, and that, in three instances, deletion of an origin selectively reduced expression of the downstream ORF-ORC gene. These data, in combination with the observation that many of the ORF-ORC genes are involved in metabolic processes, suggest a novel role for replication origins and the Origin Recognition Complex in metabolic gene regulation and, possibly, coordination of nutrient status with DNA replication.

Many of the ORF-ORC genes were identified as ORC binding sites for the first time in this study. However, several other groups, using independently generated anti-ORC antibodies, had identified one third of the ORF-ORC sites [13],[14]. Moreover, approximately thirty ORF-ORC genes also associate with Mcm2 (Table 1; [13]). One reason why these sites were not highlighted in previous ORC ChIP-on-chip studies may be that the major focus of those studies had been a comprehensive identification of replication origins, while intra-ORF ORC binding, with a few exceptions, is thought to be inconsistent with origin firing. Indeed, we found that, despite ORC and MCM binding, these sites did not function as origins of replication on the chromosome or on a plasmid (Figure 3; Table 1; Table S3). Our discovery of a large number of the ORF-ORC sites was probably due to combined sensitivity of our antibodies and our tiled arrays. Ultimately, identification of so many novel ORC sites allowed us to discern patterns and common properties and to formulate testable hypotheses regarding molecular mechanisms and functional significance.

### Transcription by RNA Polymerase II helped target ORC to protein-coding regions

Dependence on transcription, lack of a specific DNA consensus motif, and shallow and uniform shape of ORF-ORC peaks suggest that ORC binds ORF-ORC sites through a different mechanism relative to replication origins. In fact, it is likely that ORC associates with ORF-ORC sites not via direct contacts with dsDNA but through some component(s) of active transcriptional state, such as RNA, ssDNA, RNAPII apparatus, a particular nucleosome modification, or another bridging factor. Recently reported ORC interactions with histone methylase and deacetylase complexes could also be related to mechanisms underlying ORC binding within ORFs [34],[35]. It is also possible that a change in the gene's sub-nuclear localization upon transcriptional activation (e.g. re-localization to the nuclear pore) helps bring it into contact with ORC. Further research is necessary to address these issues and to distinguish which attributes of high transcription are important for attracting ORC to a gene. It is worth noting that although transcriptional state was clearly an important determinant of ORF-ORC association, even in the transcriptionally repressed state ORC association with *TDH3* was about 50% above background. Conversely, many very highly expressed genes did not interact with ORC in our ChIP assay (Figure S4). Together, these results suggest that the ORF-ORC set possesses other properties important for ORC association.

Azvolinsky et al showed that DNA polymerase and Rrm3 helicase interact with highly transcribed ORFs in transcription-dependent manner [32]. The authors proposed that this interaction could be explained by the pausing of the replisome at sites of heavy transcription by RNA Polymerase II [32]. ORC is not thought to be traveling with the replication fork [36] during S phase and yet we showed that it also interacted with many highly transcribed ORFs. These observations can be reconciled by several different non-mutually exclusive hypotheses. For instance, it is possible that ORC-ORF association and DNA polymerase-ORF association occur at different times during the cell cycle and by different mechanisms. Alternatively, origin-bound ORC could be associated with these ORFs through physical interactions with replication fork-associated proteins pausing over these ORFs. It is also possible that a fraction of cellular ORC is in fact associated with the replication fork and thus pauses over highly transcribed genes. It is worth noting here that transcription-mediated association of replication proteins with protein-coding regions has been reported in higher eukaryotes: for instance, in cultured human cells MCM proteins co-localize with RNAPII at protein-coding genes and are involved in regulating their expression [37].

### Replication origins can function to promote gene expression

It has long been observed that silent and non-silent chromatin states differ in many respects, notably origin firing and gene expression. Non-silent chromatin is characterized by early and efficient origin firing and active transcription, while silent chromatin is associated with late replication and transcriptional repression. Interestingly, while Sir proteins are primarily thought of as constituents of silent chromatin, they negatively regulate origin firing throughout the yeast genome, although molecular mechanisms of this regulation are still being elucidated [38]. Additionally, global analysis of gene expression in the *sir2*Δ mutant has implicated Sir2 in repressing transcription of amino acid biosynthesis genes [39]. Thus, silent chromatin factors have repressive effects on replication initiation and transcription within silent *and* non-silent regions. On the other hand, ORC's role in replication initiation is context-dependent. In contrast to canonical replication origins within non-silent regions, ORC's binding to silencers does not lead to efficient origin firing. Instead, it promotes formation of silent chromatin and transcriptional repression of nearby genes [20],[25]. In this report we show that deleting an origin could have the opposite effect of that expected from deleting a silencer: expression of a nearby ORF-ORC gene was reduced (Figure 7B). To our knowledge, this is the first demonstration of a replication origin acting as a positive regulator of expression of a nearby gene. Thus, we conclude that ORC's roles in *both* origin firing and gene regulation are context-dependent: within non-silent chromosomal regions ORC not only promotes efficient origin firing, but also binds to and may help induce expression of highly transcribed genes positioned nearby these origins.

### Origin position upstream of highly transcribed genes may regulate genome stability

Proximity to replication origins, as well as the directionality of this proximity, was an intriguing property of the ORF-ORC genes (Figure 6). One important aspect determined by whether a replication origin is located upstream or downstream of a gene is direction of transcription across the gene relative to direction of the replication fork. For instance, when an active origin is located closely upstream of the gene (as is the case for many ORF-ORC genes), RNA Polymerase will move in the same direction as the replication fork across the gene. It has been demonstrated that collisions between the replication fork and transcription elongation complexes moving in opposite directions lead to fork blocks and an increase in local recombination frequencies [40]. Thus, having the gene transcribed in the same direction as the movement of the replication fork reduces transcription-associated recombination [40] and may be particularly important for highly transcribed genes to reduce local replication errors. Interestingly, we found that the ORF-origin association is limited to ORC-bound genes and not generalized to all highly transcribed genes (Figure 6). Perhaps this discrepancy can be explained by differences between sets of genes highly transcribed in the laboratory relative to those highly transcribed in the wild. For instance, many ORF-ORC genes function in both metabolic and biosynthetic processes, and will thus be expressed when nutrient conditions are high (laboratory) or low (wild). On the other hand, proteins involved in ribosomal function are more highly expressed during rich conditions and fast growth [41] and may thus not be highly expressed in the wild. Thus, our current hypothesis proposes that ORC associates with a subset of metabolic genes that are highly expressed not only in the laboratory but also in the wild. These genes are frequently positioned downstream of replication origins, reducing collisions between the replication fork and transcription elongation complexes, and ORC association with these genes helps coordinate their expression with local replication dynamics.

## Materials and Methods

### Strains

All strains used in this study are of W303 background. For the ORC ChIP, CFY1211 (*ORC2*) and CFY1219 (*orc2-1*) strains were used. Both contain the synthetic *HMR*-E silencer with a high affinity binding site [23]. YPD medium (1% yeast extract, 2% peptone, and 2% glucose) was used for liquid culture growth. For experiments with the inducible *GAL1* promoter, 2% galactose was used instead of glucose. To select for uracil prototrophs during mutant construction, minimal medium supplemented with casamino acids (US Biological) was used.

Standard methods were used for yeast genetic manipulation (crosses and transformations) [42]. To create galactose-inducible *TDH3* gene, its promoter (nucleotides −650 to −1 relative to its start codon) was first replaced by the *URA3* gene. Then, *GAL1* promoter was generated by PCR with primers containing sequences targeting it to the *TDH3* locus. This PCR product was transformed into the p*TDH3*Δ*::URA3* strain and 5-FOA-resistant colonies were screened by colony PCR and verified by DNA sequencing. Deletions of *ARS731.5*, *ARS820*, and *ARS1627* were created by a similar series of transformations, except that the PCR product used to transform the *arsΔ::URA3* strains was generated by PCR fusion of two ∼200 nt stretches of DNA sequence upstream and downstream of the deleted region. To make the *ORC2-3xHA* strain, 3xHA-KANMX fragment was PCR-amplified using primers designed to target the PCR product to the *ORC2* locus by homologous recombination [43]. All transformants were verified by DNA sequencing and backcrossed once to a wild type strain. Sequences of primers used in making all of these constructs are listed in Table S4.

### Chromatin immunoprecipitation

Cultures were grown to OD_600_ of 0.5 to 0.9 and ChIPs were performed as described [44] using a cocktail of monoclonal antibodies against Orc1, Orc2, Orc3, and Orc4 proteins [45]. Average chromatin shearing size was around 0.5 Kb. IP and total DNAs were purified using a QIAquick PCR purification kit (Qiagen). Quantification of DNA amounts was done in one of the following two ways. For results shown in Figure 1A, Figure 2C, and Figure 7B (*ARS731.5* and *TDH3* loci) appropriately diluted IP and total DNA samples were subjected to 26 cycles of PCR using gene-specific primers (Table S4), the PCR products were separated on a 1.25% agarose gel containing GelRed dye (Biotium), and band intensities were quantified using video densitometry analysis and Labworks analysis software (UVP). For results shown in Figure 2B and 2C and Figure 7B (*ARS820*, *ENO2*, *ARS1627*, and *ASN1* loci) quantitative real time PCR reactions containing the SYBR Green Power Mix (Applied Biosystems) and gene-specific primers (Table S4) was performed and reaction products analyzed by SDS software (Applied Biosystems). For the HA ChIP, cell lysates were incubated with a mouse monoclonal anti-HA antibody (Santa Cruz Biotechnologies), followed by immunoprecipiation with Protein G Dynabeads (Invitrogen), and analyzed by real-time PCR as described above. All PCR reactions were performed in duplicate, with at least two biological replicates analyzed for each genotype. Since ORC ChIP-on-chip showed no binding of ORC to the *FKH1* gene, *FKH1* PCR was performed in every instance to measure non-specific/background ORC binding (with the exception of experiment shown in Figure 1A where *ADH4* was used for this purpose). Thus, for each sample, its IP/total ratio was determined and normalized to that of *FKH1*.

For ORC ChIP-on-chip, cells were grown at 23°C, the permissive temperature for *orc2-1*. Under these conditions, Orc2p levels are reduced approximately 10-fold compared to wild type Orc2p [28]. IP and total DNA were amplified using ligation-mediated PCR and mailed to Nimblegen for hybridization to high density tiled arrays (2006-10-12_Ansari_tiling_51mer). For each ChIP-on-chip experiment, the immunoprecipitated (IP) sample was labeled with Cy3 and the input (sheared genomic DNA) was labeled with Cy5. The log_2_ ratios of IP over input were obtained from the values extracted from the *S. cerevisiae* tiling microarray for each feature. These log_2_ ratios for each experiment were plotted as a histogram and then background subtracted so that the peak of each histogram was centered over 0. The most repetitive probes (1.1%) were removed from the dataset. Peaks were identified by ChiPOTle [46] assuming a Guassian distribution, and using a window size of 400 and step size of 100. Peak area was calculated as the sum of the log_2_ ratios of all probes that were contained within the peak. The p-value cut-off of 10^−20^ was judged to give the best compromise between retaining as many confirmed ORC peaks as possible while removing small peaks that may be due to non-specific or artifactual binding (Figure S2).

RNA Polymerase II (RNAPII) ChIP was performed with an antibody against RNAPII (αRPB3) as described previously [47]. The ChIP samples were amplified using ligation-mediated PCR and hybridized to high density tiling microarrays from NimbleGen (2006-10-12_Ansari_tiling_51mer).

### Data deposition

The ORC ChIP-chip data are available both from Gene Expression Omnibus (http://www.ncbi.nlm.nih.gov/geo) and the Origin Database (www.oridb.org).

### Bioinformatics

Calculation of ORF-ORC peak distances and ORF-ARS distances was done using PERL scripts specifically written for this purpose and available upon request. Searches for consensus motifs within and around ACS elements and ORF-ORC sequences were done using MEME (http://meme.sdsc.edu). To determine whether the ORF-ORC set was enriched in certain functional categories, we used the *Saccharomyces* Genome Database (SGD) Gene Ontology (GO) Term finder (http://www.yeastgenome.org/cgi-bin/GO/goTermFinder.pl). Verified and uncharacterized genes from the ORF-ORC set were submitted as a query against all *S. cerevisiae* verified and uncharacterized ORFs. Categorization of genes by functional process (Figure 5) was done according to SGD GO term annotation.

### Two-dimensional gel electrophoresis

2D gel origin assays were performed as previously described (Friedman and Brewer 1995). Primers used to generate probes are listed in Table S4.

### Plasmid ARS assay

A recombination-dependent ARS assay was used as before [48]. Genomic DNA fragments of interest were cloned within lacZ sequences or selected from a genomic library [49]. Clones were tested for ARS activity by co-transformation into yeast with a linearised vector (YCplacZ) that lacks an ARS [50]. Colonies are only observed if the genomic DNA fragment contains an ARS.

### Gene expression analysis

For global gene expression analysis, total RNA samples were obtained as described previously [47] and were labeled and hybridized to high density tiling microarrays by NimbleGen. Probe intensities were divided by the peak intensity from the raw data histogram and then Log_2_ transformed.

To determine expression levels of individual genes, total RNA was prepared from cells grown to an OD_600_ of 0.5 using either standard hot phenol extraction methods or the RNeasy kit (Qiagen). Total RNA concentration was determined by spectroscopic analysis using NanoDrop (Thermo Scientific) and/or agarose gel electrophoresis. For reverse transcription (RT), 1–5 µg of total RNA was used in a 20-µl reaction mixture using oligo(dT)_12–18_ primers (Invitrogen) and Superscript III (Invitrogen) following the manufacturer's protocol. Relative amounts of cDNAs of various genes were measured either by PCR followed by band densitometry (Figure 5B, Figure 7B – *TDH3*) or by SYBR Green real time PCR (Figure 7B – *ENO2* and *ASN1*) as described above for ChIPs. Expression of every gene was normalized to expression of *ACT1* from the same RNA preparation. Most strains analyzed were *MAT*α.

## Supporting Information

Figure S1 A genome-wide look at our ORC ChIP-on-chip results. In the top panel of each chromosome, log ratios of immunoprecipitate over total chromatin for *ORC2* (black) and *orc2-1* (blue) strains are averaged over 1 Kb segments and plotted against chromosomal coordinate. Segments are connected via their midpoints (e.g. 500, 1,500, 2,500, etc) and smoothed. Please note that the scale is different for each chromosome. The horizontal black line through the *ORC2* and *orc2-1* data represents a log ratio of zero. The bottom panel displays the ARS regions on each chromosome as defined by the Replication Origin Database (www.oriDB.org). The majority of our ORC peaks correspond to previously identified sites of ORC binding and/or origin firing. On the OriDB panel, narrow peaks usually correspond to “confirmed” ARSs that have been defined to within a few hundred basepairs, while ARS segments that are one or more kilobases in length usually correspond to “likely” or “dubious” ARSs.
(1.07 MB PDF)

Figure S2 ORC peaks with a p-value of 10^−20^ or better were chosen for further analysis. This figure shows an example of wild type ORC trace over a region of chromosome 15. Peaks in solid line boxes had been assigned p-values of 10^−20^ or better (lower) by Chipotle software and were analyzed further. They include a “confirmed” ARS, a “likely” ARS, and a novel ORC site. Peaks in dashed line boxes were assigned a p-value higher than 10^−20^ were deemed too weak/insignificant to warrant further study.
(3.97 MB TIF)

Figure S3 ORC and MCM associate with *ENO2* ORF in a different ChIP-on-chip. A screen capture from OriDB (http://www.oridb.org/charts/graphic.php?id=700&view=default) is showing origin summary graphics at the region encompassing “confirmed” *ARS820* and “likely” *ARSVIII-452 (ENO2)*. Blue bars indicate Mcm2 binding and green bars indicate ORC binding [14].
(10.24 MB TIF)

Figure S4 Many highly expressed genes did not associate with ORC *in vivo*. Top 10% highest expressed genes were compared to the ORF-ORC gene set, showing that many highly expressed genes did not show ORC binding *in vivo*.
(3.89 MB TIF)

Table S1 ChIP-on-chip overview.
(0.18 MB XLS)

Table S2 ORF-ORC sites.
(0.08 MB XLS)

Table S3 Full ARS assay results.
(0.04 MB XLS)

Table S4 Primer list.
(0.04 MB DOC)
