# Supplementary material for: The Origin Recognition Complex Interacts with a Subset of Metabolic Genes Tightly Linked to Origins of Replication
Source: PLoS Genet. 2009 Dec 4;5(12):e1000755. doi: 10.1371/journal.pgen.1000755 (PMC2778871; doi:10.1371/journal.pgen.1000755)
Supplement: Figure S1 — A genome-wide look at our ORC ChIP-on-chip results. In the top panel of each chromosome, log ratios of immunoprecipitate over total chromatin for ORC2 (black) and orc2-1 (blue) strains are averaged over 1 Kb segments and plotted against chromosomal coordinate. Segments are connected via their midpoints (e.g. 500, 1,500, 2,500, etc) and smoothed. Please note that the scale is different for each chromosome. The horizontal black line through the ORC2 and orc2-1 data represents a log ratio of zero. The bottom panel displays the ARS regions on each chromosome as defined by the Replication Origin Database (www.oriDB.org). The majority of our ORC peaks correspond to previously identified sites of ORC binding and/or origin firing. On the OriDB panel, narrow peaks usually correspond to “confirmed” ARSs that have been defined to within a few hundred basepairs, while ARS segments that are one or more kilobases in length usually correspond to “likely” or “dubious” ARSs. (1.07 MB PDF) [file pgen.1000755.s001.pdf]

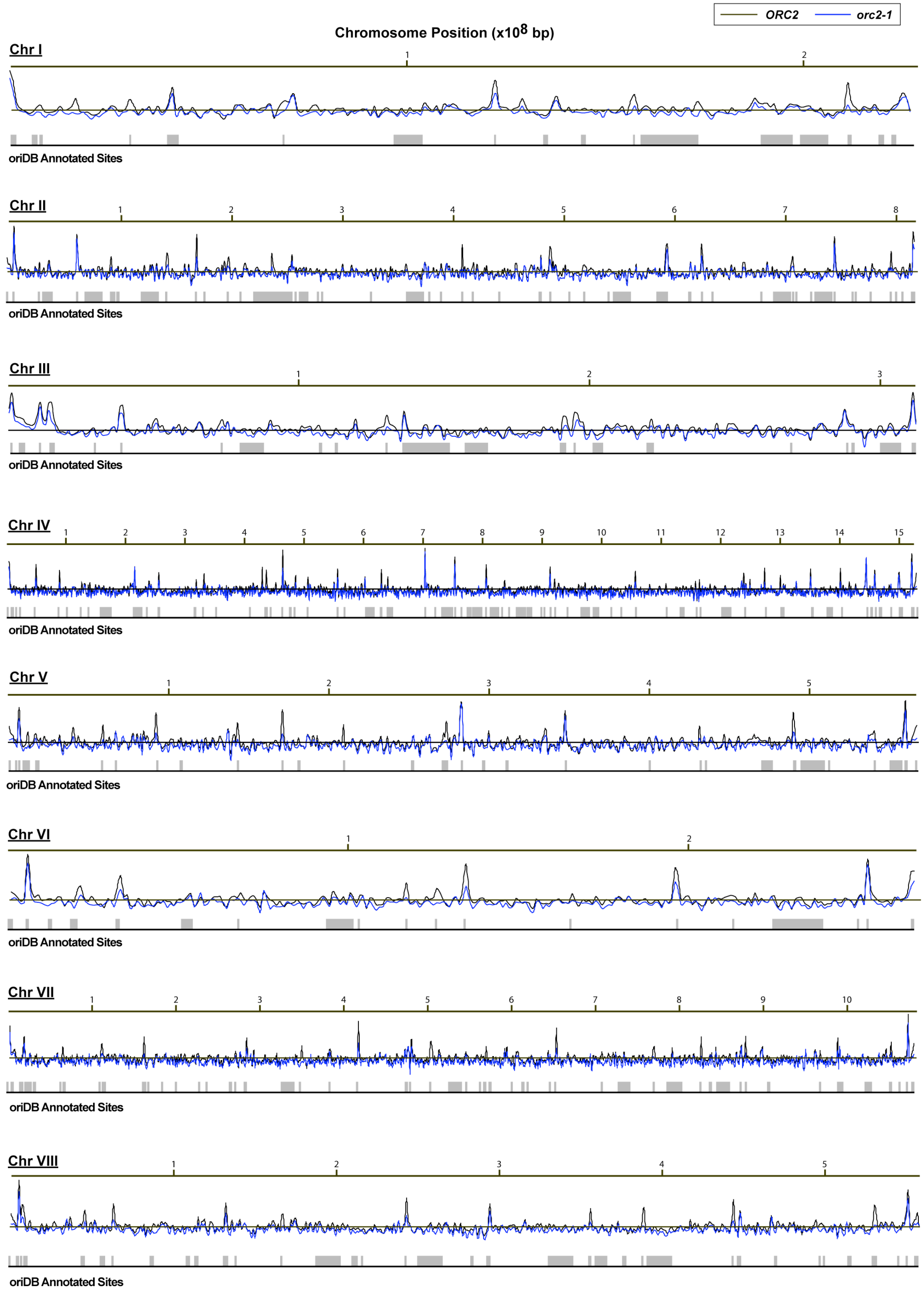
ORC2
orc2-1
Chromosome Position (x10^8 bp)
Chr I
1
2
oriDB Annotated Sites
Chr II
1
2
3
4
5
6
7
8
oriDB Annotated Sites
Chr III
1
2
3
oriDB Annotated Sites
Chr IV
1
2
3
4
5
6
7
8
9
10
11
12
13
14
15
oriDB Annotated Sites
Chr V
1
2
3
4
5
oriDB Annotated Sites
Chr VI
1
2
oriDB Annotated Sites
Chr VII
1
2
3
4
5
6
7
8
9
10
oriDB Annotated Sites
Chr VIII
1
2
3
4
5
oriDB Annotated Sites

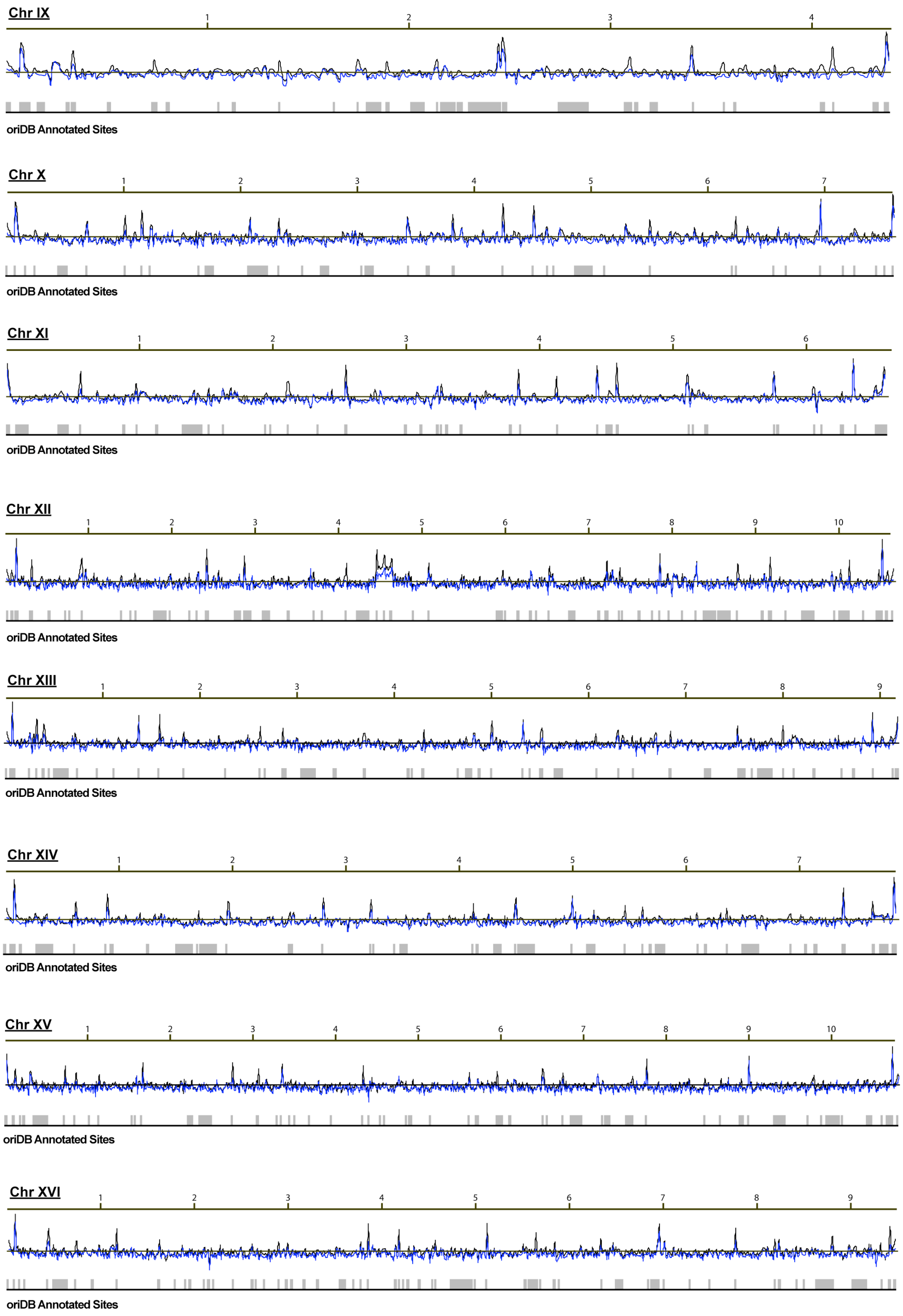
Chr IX
1 2 3 4
oriDB Annotated Sites
Chr X
1 2 3 4 5 6 7
oriDB Annotated Sites
Chr XI
1 2 3 4 5 6
oriDB Annotated Sites
Chr XII
1 2 3 4 5 6 7 8 9 10
oriDB Annotated Sites
Chr XIII
1 2 3 4 5 6 7 8 9
oriDB Annotated Sites
Chr XIV
1 2 3 4 5 6 7
oriDB Annotated Sites
Chr XV
1 2 3 4 5 6 7 8 9 10
oriDB Annotated Sites
Chr XVI
1 2 3 4 5 6 7 8 9
oriDB Annotated Sites
