# Supplementary figures and images for: The Origin Recognition Complex Interacts with a Subset of Metabolic Genes Tightly Linked to Origins of Replication
Source: PLoS Genet. 2009 Dec 4;5(12):e1000755. doi: 10.1371/journal.pgen.1000755 (PMC2778871; doi:10.1371/journal.pgen.1000755)

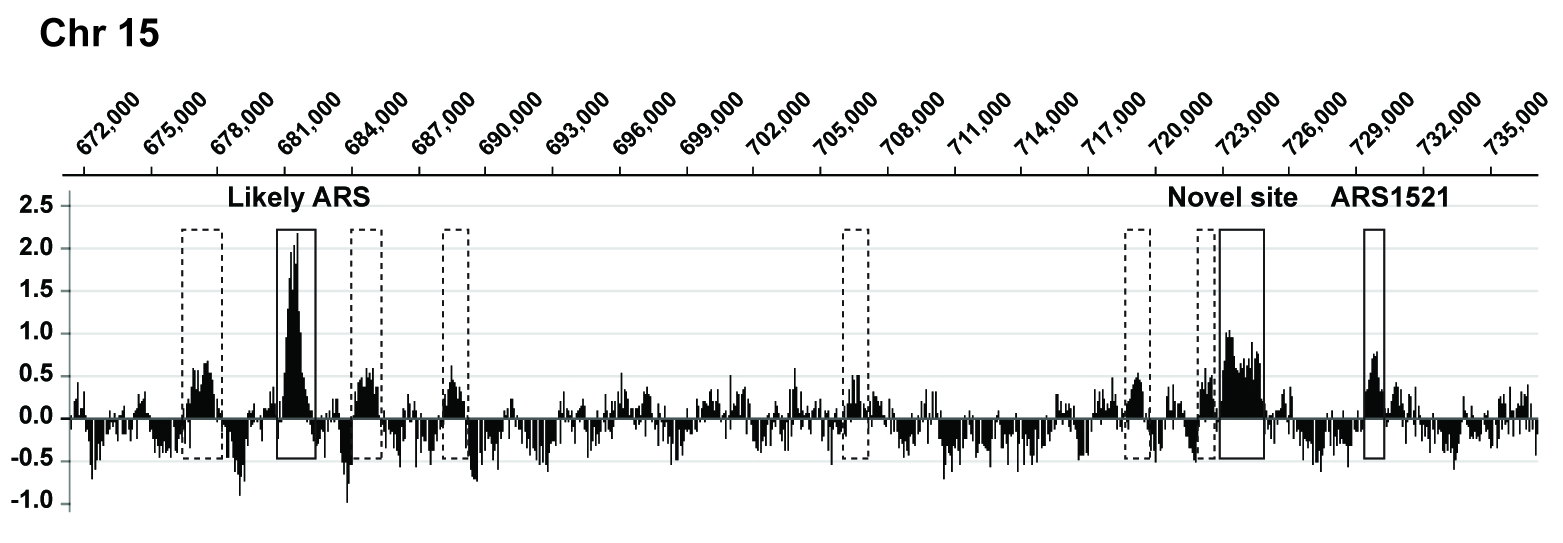

Supplement: Figure S2 — ORC peaks with a p-value of 10−20 or better were chosen for further analysis. This figure shows an example of wild type ORC trace over a region of chromosome 15. Peaks in solid line boxes had been assigned p-values of 10−20 or better (lower) by Chipotle software and were analyzed further. They include a “confirmed” ARS, a “likely” ARS, and a novel ORC site. Peaks in dashed line boxes were assigned a p-value higher than 10−20 were deemed too weak/insignificant to warrant further study. (3.97 MB TIF) [file pgen.1000755.s002.tif]

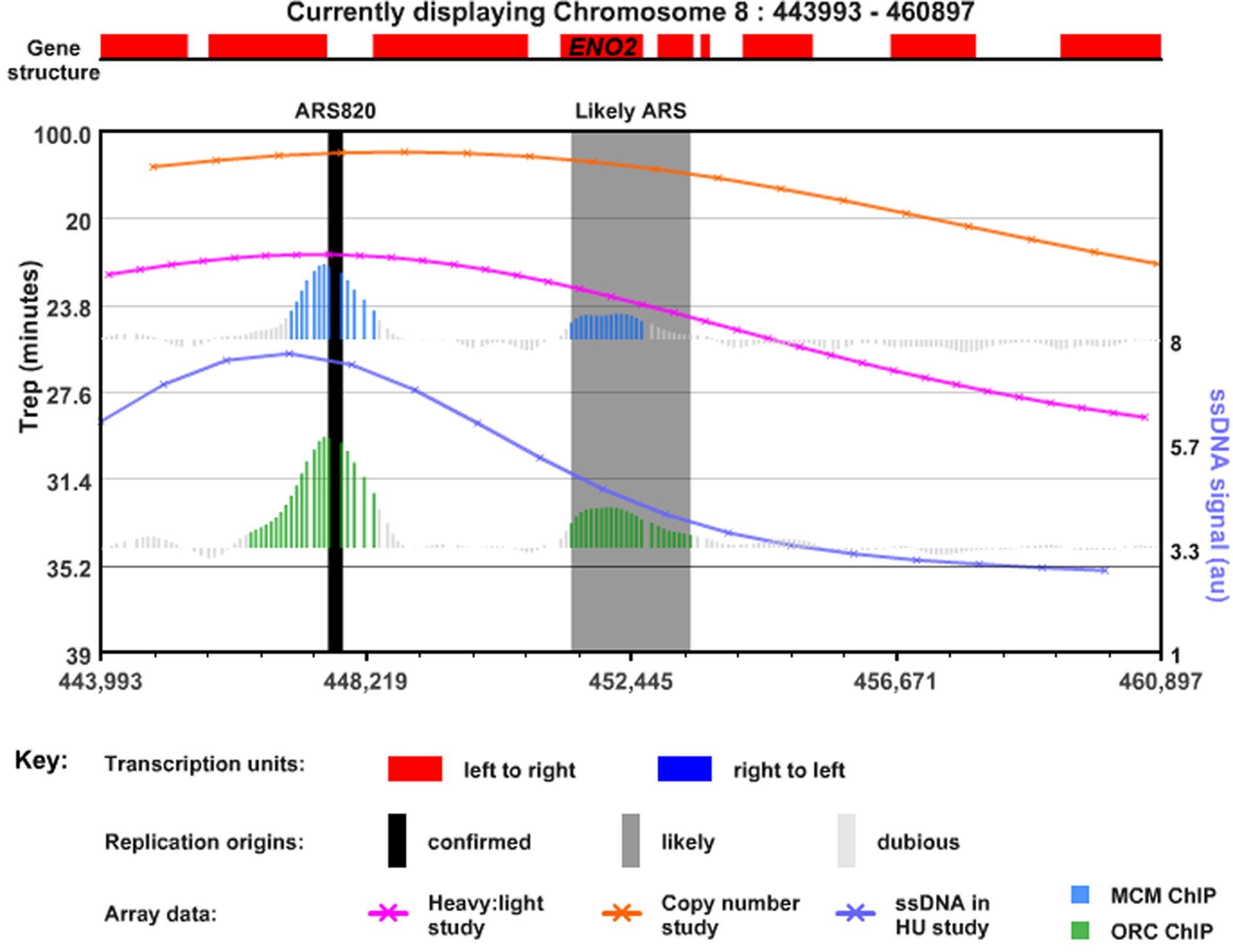

Supplement: Figure S3 — ORC and MCM associate with ENO2 ORF in a different ChIP-on-chip. A screen capture from OriDB (http://www.oridb.org/charts/graphic.php?id=700&view=default) is showing origin summary graphics at the region encompassing “confirmed” ARS820 and “likely” ARSVIII-452 (ENO2). Blue bars indicate Mcm2 binding and green bars indicate ORC binding [14]. (10.24 MB TIF) [file pgen.1000755.s003.tif]

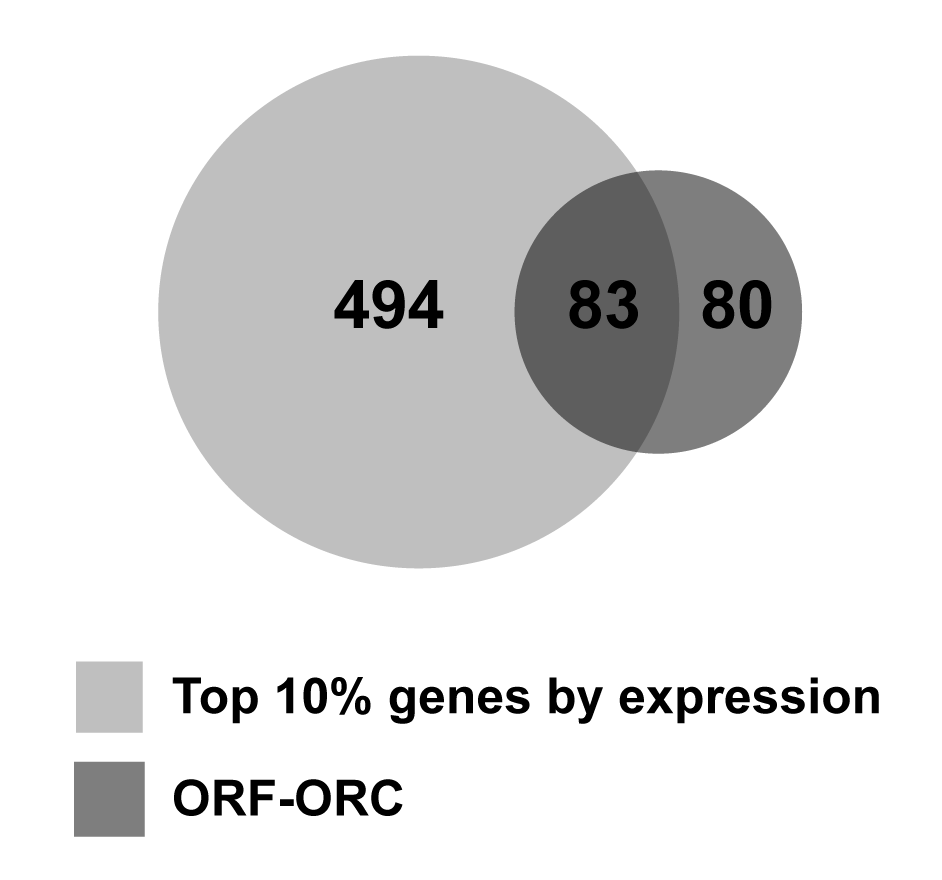

Supplement: Figure S4 — Many highly expressed genes did not associate with ORC in vivo. Top 10% highest expressed genes were compared to the ORF-ORC gene set, showing that many highly expressed genes did not show ORC binding in vivo. (3.89 MB TIF) [file pgen.1000755.s004.tif]
