# Supplementary material for: The Origin Recognition Complex Interacts with a Subset of Metabolic Genes Tightly Linked to Origins of Replication
Source: PLoS Genet. 2009 Dec 4;5(12):e1000755. doi: 10.1371/journal.pgen.1000755 (PMC2778871; doi:10.1371/journal.pgen.1000755)
Supplement: Table S4 — Primer list. (0.04 MB DOC) [file pgen.1000755.s008.doc]

- **2D gel probe primers:**

Likely ARSVII-883 (TDH3): ACGGGTCTTACTGATGAGGC & TCAATGCCTGCAATGTCTCC

- ARS731.5: AAAGGTCCAAAAGAGCCAAG & GGGGCAGCAGAATAACTTAC
- ARS820: CAAGGGCGTATGAAAAATGG & TTGTTTGGATTTTCGGATTG
- Likely ARSVIII-452 (ENO2): CCTTCCCTTTATTTGTTCCC & CGGTGCTGAAATGGATGCTG
- ARS1005: TATGTTGGTAAAATGTCGGG & GAAAGAATCGCAAACGGAGTG
- ARS1006: TATTATGGCTGCTTCCTTCG & AATCGTGCGTTTCAAGTGTC

**ChIP/RT-PCR primers:**

ADH4: ggctactaacggtggggaaatcggagac & gcacaggcatcggtgattgggttagaggc

HMRss: ggtagagttccttgttgaacgtgataaccc & cgcctaccttcttgaacaagatggatagc

Natural HMR: ATAAAATCCTCGCACTATCGCTG & GCAAATGTGGACGAAAAGAAATGC

ARS1: ggcgttattggtgttgatgtaagcggagg & gcaagaccgagaaaaggctagcaagaatcg

FKH1: CGGGTGTTGAAAGAATGGCTTGGG & GCCTGAATCACCTCATGCCCAAATTAC (for densitometry)

CCCAAGCCATTCTTTCAACACC & AATCACGCCTGACTTCATCGC (for real time PCR)

ACT1: caagaaatgcaaaccgctgc & ggtcaataccggcagattcc

TDH3: ATCACCAACGACTACGCTGC & TGAGTAGCAGTCAAAGAGTGGACAG

ENO2: GAAAAGAACGTCCCATTGTACC & CTTGGATTTGTCAGATTCTGGG (for densitometry)

- CGAAAAGAAGGCTGCTGACG & GAAACCATAACACCCCAGTTGG (for real time PCR)
- ASN1: TTGCTTGGACTCTTTACGACG & AGTCCAAACCACCCGACAATAG

GAL1: gcggtgaggaagatcatgctctatacgttg & ccagggtgtggaaatgttgtgatatctggc

PDX1: TCAATGCCTGCAATGTCTCC & GGATGGTAATAGCGTCTGTTCAAG

HIP1: GTTCTCTTTGCTGCCTTGGG & CCTGGGTTGCTATCTTTTATGC

ARS731.5: TTGCTGGTTGGCTTTCGTAG & TGGAATAATGGTCTTGATCCCC

ARS820: TCAGTCATAAAGCAGGAGGTCG & CTTAGCCGTCAGCAACGAAC

SPC97: GTTGAAAGGCTTGAAAGGGAC & AAACATTACGCTTGACCTATCACC

CTR2: CAAGATGGAAGGAAATGCGG & TATTGCCCCACACACGACAG

FMO1: AAGAGCCTGCGAGCAATCAAC & GCACTTCCAGTCTAACGGAGGG

ARS1627: CTATGTCAAGCGAGAAAGGGG & CACGGTGCCTTAACCAACTG

KAR3: CGCTGTGTTTTGTATTTTCGTCAC & CAAGGCTGAGAGATGAGGTGG

RRP15: GGTTCTACTGGATTTTCTGCTGC & TCGGATACTTCCTTTTCTGTCTTG

NOC4: TCATCAACACATTTATCCGCCC & TCCCACAGAGAGGAATCCAGTG

**Primers to make p*GAL1-TDH3*:**

To amplify p*TDH3∆::URA3*:

- GAATAAAAAACACGCTTTTTCAGTTCGAGTTTATCATTATCAATACTGCC**GAGTGCACCATACCACAGC**
- &
- ATGACCAATCTACCGATTCTACCGAAACCGTTAATAGCAACTCTAACCAT**TGTGCGGTATTTCACACC**

To amplify p*GAL1* with regions of *TDH3* homology:

- GAATAAAAAACACGCTTTTTCAGTTCGAGTTTATCATTATCAATACTGCC**AAAGAGCCCCATTATCTTAG**

&

ATGACCAATCTACCGATTCTACCGAAACCGTTAATAGCAACTCTAACCAT**GGGTTTTTTCTCCTTGACG**

**Primers to make *ARS731.5∆*:**

- To amplify *ars731.5∆::URA3*: TATTCCAGTGAATGATCTACTTTTGCTTAAGCGGCAGAATTGCAAATCTT**GAGTGCACCATACCACAGC**
- & CATTTCTATCAGTTTCTATACCAAAAGAAGAAAAAGTGGAGAATTGTTTG**TGTGCGGTATTTCACACC**

To amplify regions left and right of the deletion, followed by fusion PCR to create the *ars731.5∆* fragment:

- 1. ACAATGGTTCGCAATGTC
- 2. AAAAGTGGAGAATTGTTTGT**AAGATTTGCAATTCTGCC**
- 3. ACAAACAATTCTCCACTTTTTCTTC

4. TGGAATAATGGTCTTGATCCCC

**Primers to make *ARS820∆*:**

- To amplify *ars820ACS∆::URA3*: TACATTATTTTTTGTAGTTTTAACAAGAAGACAAATTCTTGACTTTACTC**GAGTGCACCATACCACAGC**
- &
- CTGATCCTTTTTGAAAAATACGGTGAACATTGAGGACTTTATTACATTTT**TGTGCGGTATTTCACACC**

To amplify regions left and right of *the deletion*, followed by fusion PCR to create the *ars820ACS∆HindIII* fragment (the HindIII site is in bold):

- 1. TCAGTCATAAAGCAGGAGGTCG
- 2. GAGGACTTTATTA**AAGCTT**GAGTAAAGTCAAGAATTTGTC
- 3. CTTTACTC**AAGCTT**TAATAAAGTCCTCAATGTTCACCG

4. CTTAGCCGTCAGCAACGAAC

**Primers to make *ARS1627∆*:**

- To amplify *ars1627ACS∆::URA3*:

ATACAGGTGTGGTGTCCTTCACCATCGATATATTAATATTAATATATCAT**GAGTGCACCATACCACAGC**

&

- CTAAGGGAAATTGCTATATTCTAAAAGAAGTGCACAAGTACATTACCTTG**TGTGCGGTATTTCACACC**

To amplify regions left and right of *the deletion*, followed by fusion PCR to create the *ars1627ACS∆HindIII* fragment (the HindIII site is in bold):

1. CTATGTCAAGCGAGAAAGGGG

- 2. AAGTACAT**AAGCTT**GATGATATATTAATATTAATATATCG
- 3. ATCATC**AAGCTT**ATGTACTTGTGCACTTCTTTTAG

4. CACGGTGCCTTAACCAACTG

**Primers to amplify the *ORC2-3xHA-KANMX* construct using the Pringle-Longtine method:**

GGAACTTGAAAAACTTCTGAAAACCGTTTTAAATACTCTAcggatccccgggttaattaa

&

AAGCCTAGTACTATTACAATTGTTCGTGATATGTATACATgaattcgagctcgtttaaac
